# Supplementary material for: Spatiotemporal functional organization of excitatory synaptic inputs onto macaque V1 neurons
Source: Nat Commun. 2020 Feb 4;11:697. doi: 10.1038/s41467-020-14501-y (PMC7000673; doi:10.1038/s41467-020-14501-y)
Supplement: Supplementary file 1 — Supplementary Information [file 41467_2020_14501_MOESM1_ESM.pdf]

Supplementary information

Spatiotemporal functional organization of excitatory synaptic inputs onto macaque V1 neurons

Ju et al.

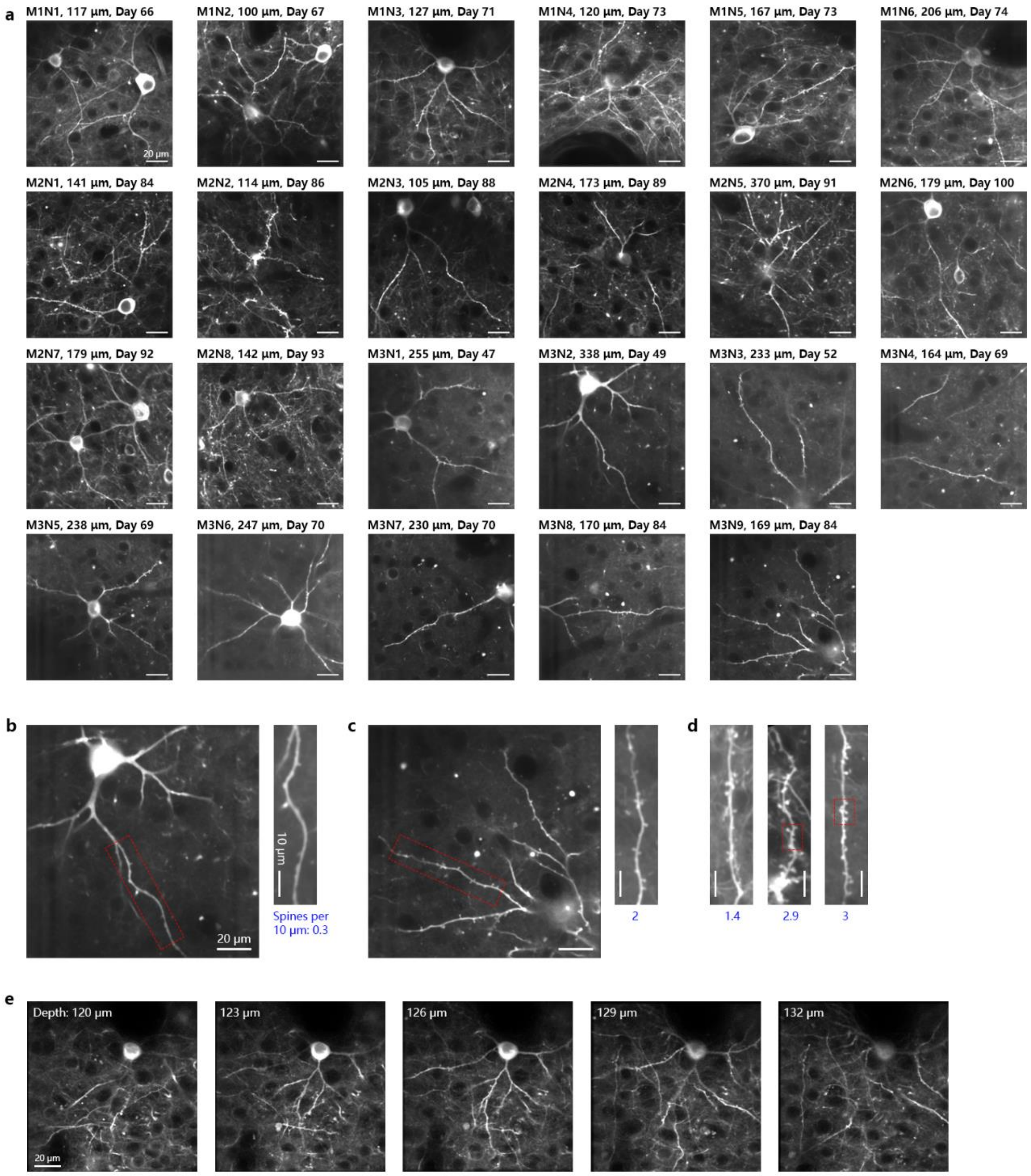

Supplementary Figure 1. Sparse labeling of iGluSnFR on macaque V1. a Two-photon images (1000 frames averaged from each recording session) from all neurons reported in this study (23 neurons recorded from 3 monkeys). The AAV1.hSynap.SF-iGluSnFR.A184S.WPRE.SV40 serology/promoter/construct combination resulted in sparse labeling in macaque V1. Panel titles denote recording index, depth of recording and the date of the recording with respect to the number of days post-infection. **b** Left, an enlarged image of neuron M3N2

reveals smooth dendrites with few spines. Right, magnified field of view (red box in the left panel). Average spine density indicated in blue. **c** Neuron M3N9, with spiny dendrites, presented in the same format as panel **b**. **d** Spiny dendrites from three other neurons (M1N5, M2N2 and M3N5). Dense spine distributions were found in several dendritic branches (red boxes), with up to 5 spines per 10 μm. **e** Averaged images obtained with a 25x objective lens under zoom 1x, covering a field of view of 128 μm, averaged across 100 frames each, recorded at a 3 μm depth interval from 120 μm to 132 μm.

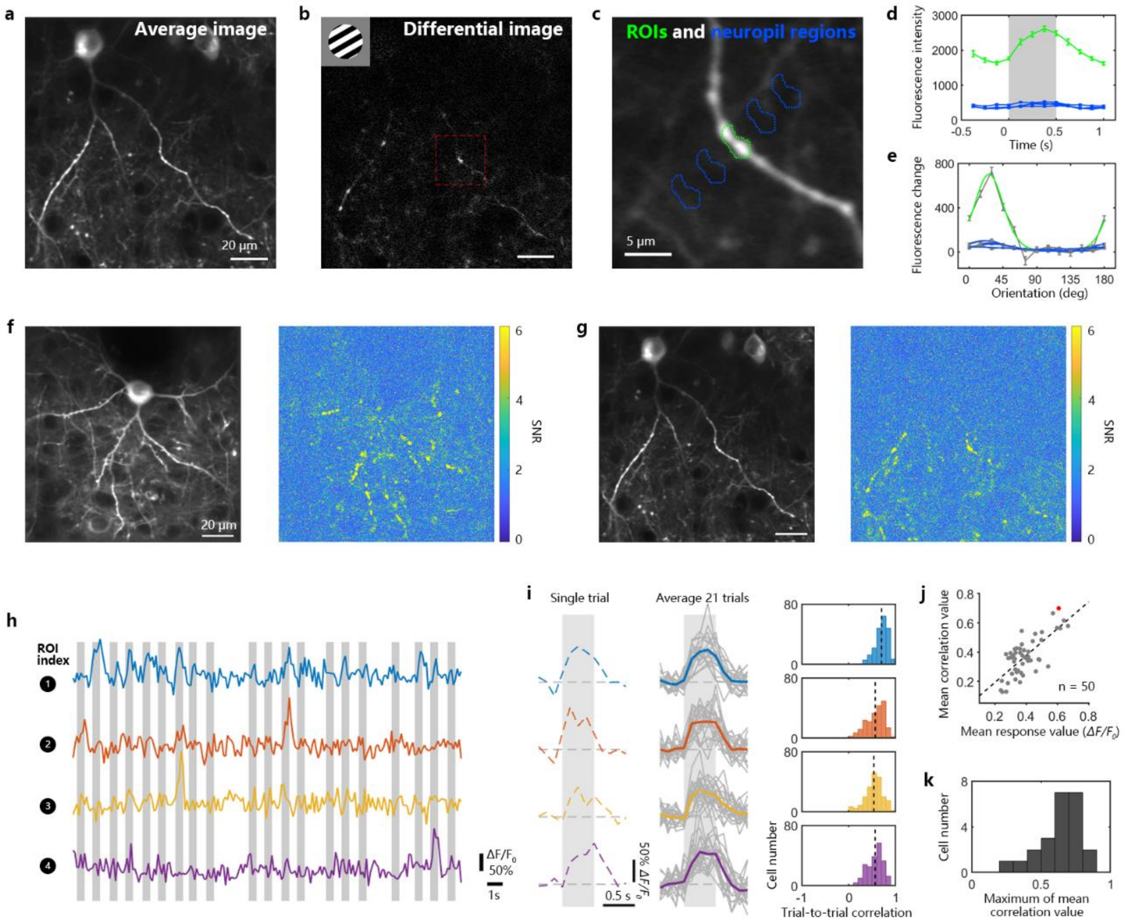

Supplementary Figure 2. Trial-to-trial iGluSnFR response analysis. a A sparsely labeled neuron's (depth of 105 μm on day 88 post-injection) average image from the OFF period of a specific stimulus condition (taken the 4 frames before stimulus onset, with 21 repeats). The neuron and its dendrites are well isolated from the neuropil in the background due to spare labeling. **b** The difference image created from ON and OFF stimulus periods (ON-OFF) exhibits hotspots on target dendrites. **c** A magnified FOV of one target ROI and its neighboring neuropil regions, as control. **d** Fluorescence intensities (averaged across 21 repeats) of the target ROI (green line) and control regions (blue lines) along the presentation of the stimulus as shown in **b**. Grey shaded area denotes the stimulus onset period. Error bar, SE. **e** Mean fluorescence changes (ON period

– OFF period) to 12 different orientations show clear orientation tuning at target ROI site. **f-g** SNR maps of iGluSnFR fluorescence signals. Left, two-photon image of an example neuron at a depth of 127 μm, averaged across 1000 frames from one recording session. Right, pixel-by-pixel SNR map of fluorescence signals under the same field of view. SNR value is denoted by the colorbar on the right side. Right, same for another sample neuron at a depth of 105 μm. **h** Raw traces of iGluSnFR fluorescence from 4 sample ROIs (from **Fig. 1a**) during several consecutive stimulation conditions in one recording session. **i** Single-trial fluorescence transients from the 4 ROIs stimulated by their preferred stimuli. Left, single trials; middle, average (colored) of 21 individual trials (gray); right, distribution of trial-to-trial correlations of time courses (blue, 0.70 ± 0.14; orange, 0.57 ± 0.19; yellow, 0.54 ± 0.19; purple, 0.56 ± 0.17; mean $\pm$ STD, $n = 21$ trials). **j** Mean trial-to-trial correlation value versus mean response value for all 50 ROIs on the target neuron dendrites. Red dot, the ROI with the highest mean correlation value; black dash line, best-fit linear regression line ($p = 9 \times 10^{-10}$). **k** Statistics of the highest mean correlation value for each neuron ($n = 23$).

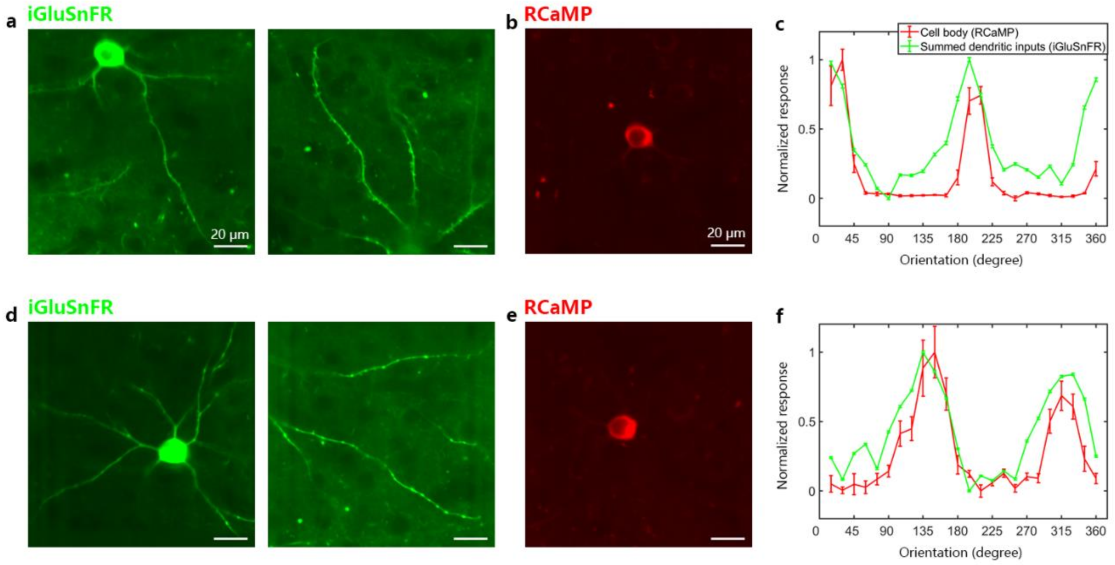

Supplementary Figure 3. Co-expression of RCaMP and iGluSnFR in individual neurons. a Two-photon images (averages of 1000 frames) of iGluSnFR fluorescence were acquired using a 1000 nm excitation laser and a 525 ± 35 nm emission filter. The two fields of view show different dendritic regions (at depths of 233 μm and 250 μm respectively) of one sample neuron (day 52 post-infection). Scale bar, 20 μm. **b** Two-photon images of RCaMP fluorescence of the same neuron as shown in **a** were acquired with a 1060 nm excitation laser and a 615 ± 20 nm emission filter, by averaging 1000 frames from each recording session. This recording was made at a depth of 233 μm. **c** Normalized responses of RCaMP signals (red line), and summed iGluSnFR signals of target ROIs (green line). Error bar, SE across 20 trials. **d-f** Same as **a-c**, for another sample neuron (depth of 247 μm on day 70 post-infection).

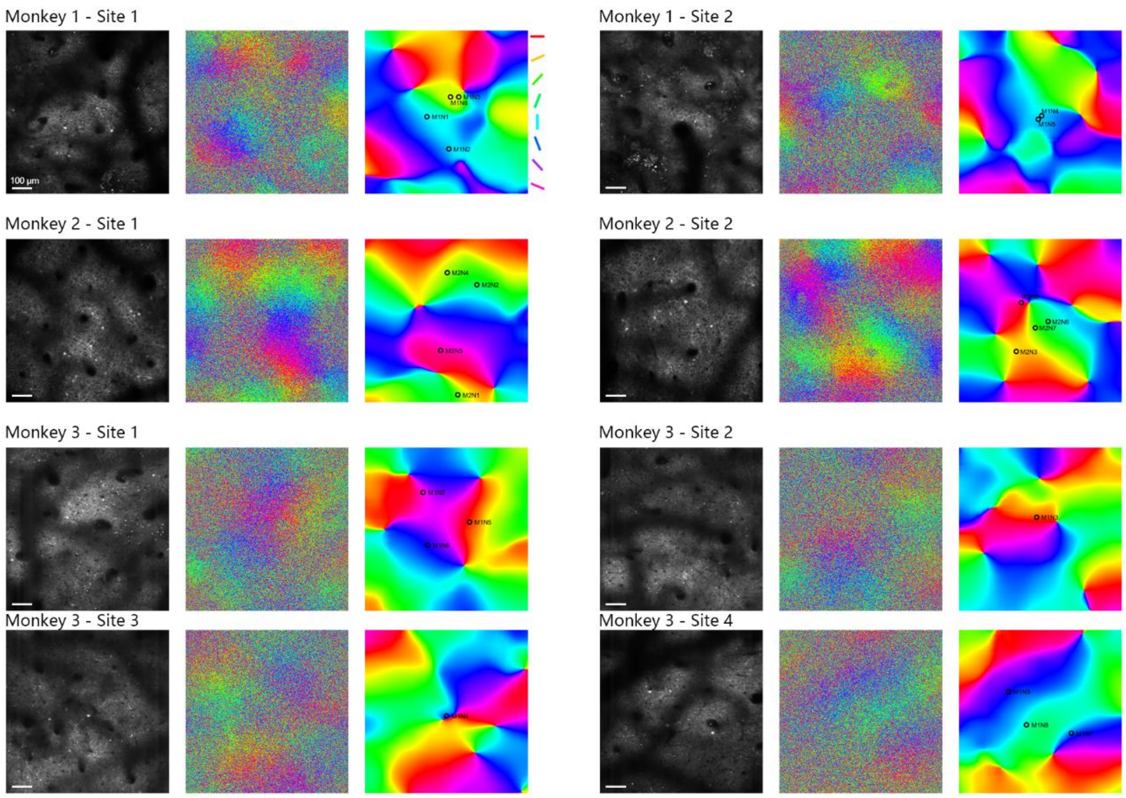

Supplementary Figure 4. Orientation maps for each imaging site from 3 monkeys. For each imaging site, left panel: an averaged image from all frames acquired during corresponding recording session; middle: pixel-by-pixel orientation map without smoothing; right: orientation map after Gaussian filtering, black circles represent the cortical position of each target neuron.

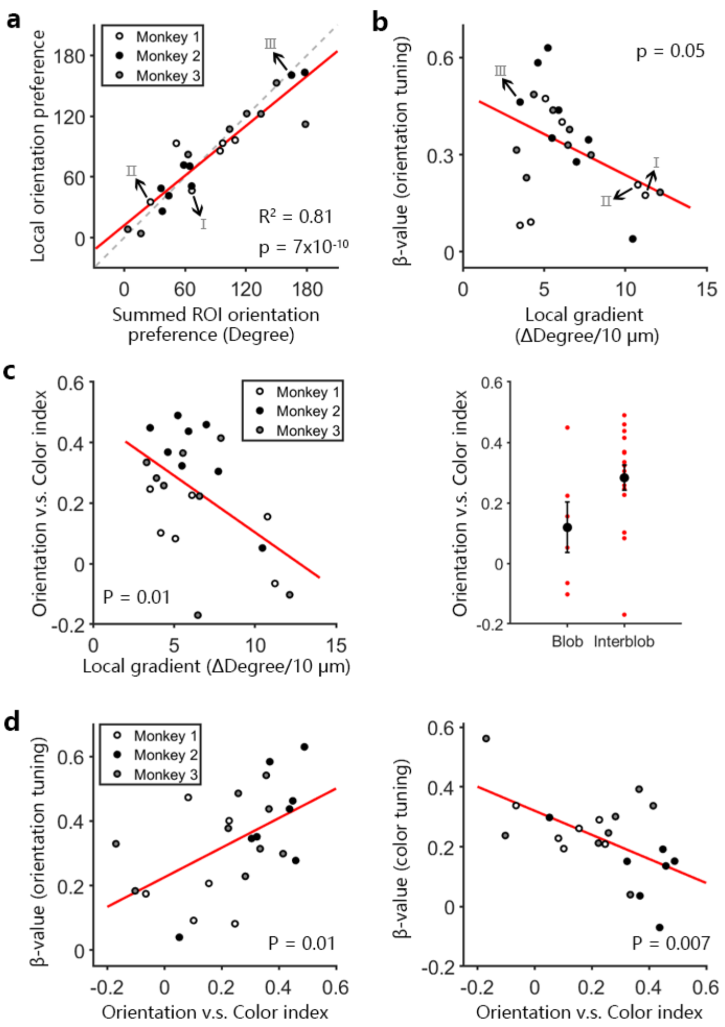

Supplementary Figure 5. Position-dependence of orientation vs color dominance within the cortical orientation map. a Local cortical orientation preference versus that of summed dendritic ROI responses for each single neuron. Red line, regression line; grey dashed line, unity line. $R^2 = 0.81$ when fitted to the unity line. **b** β-value of dendritic orientation tuning versus local gradient of orientation preference. **c** Left, orientation versus color index as a function of the local gradient of orientation selectivities of each neuron ($n = 22$). Red line, linear fit ($y = kx + b$, $k = -0.038 \pm 0.028$, $b = 0.48 \pm 0.20$; 95% condifence intervals; $p = 0.01$); white dots, neurons from Monkey 1; black, Monkey 2; grey, Monkey 3. Right, relationship between the average orientation versus color index and the

position of the neurons within the orientation-selectivity map. Red dots represent individual neurons ($n = 22$); error bar, SEM. The results demonstrate that neurons with regions of high orientation-selectivity (i.e. interblobs) tend to receive more orientation-selective inputs, whereas neurons in regions of low orientation-selectivity (i.e. blobs) tend to receive more color-selective inputs. **d** Left, orientation correlation baseline (β-value) as a function of the orientation versus color index for each neuron ($n = 23$, $k = 0.46 \pm 0.33$, $b = 0.23 \pm 0.10$; $p = 0.01$). Right, color correlation baseline as a function of the orientation versus color index ($n = 22$; $k = -0.40 \pm 0.28$, $b = 0.32 \pm 0.08$; $p = 0.007$). The high correlations suggest that the proportion of inputs of a given type specifies the baseline function of the neuron.

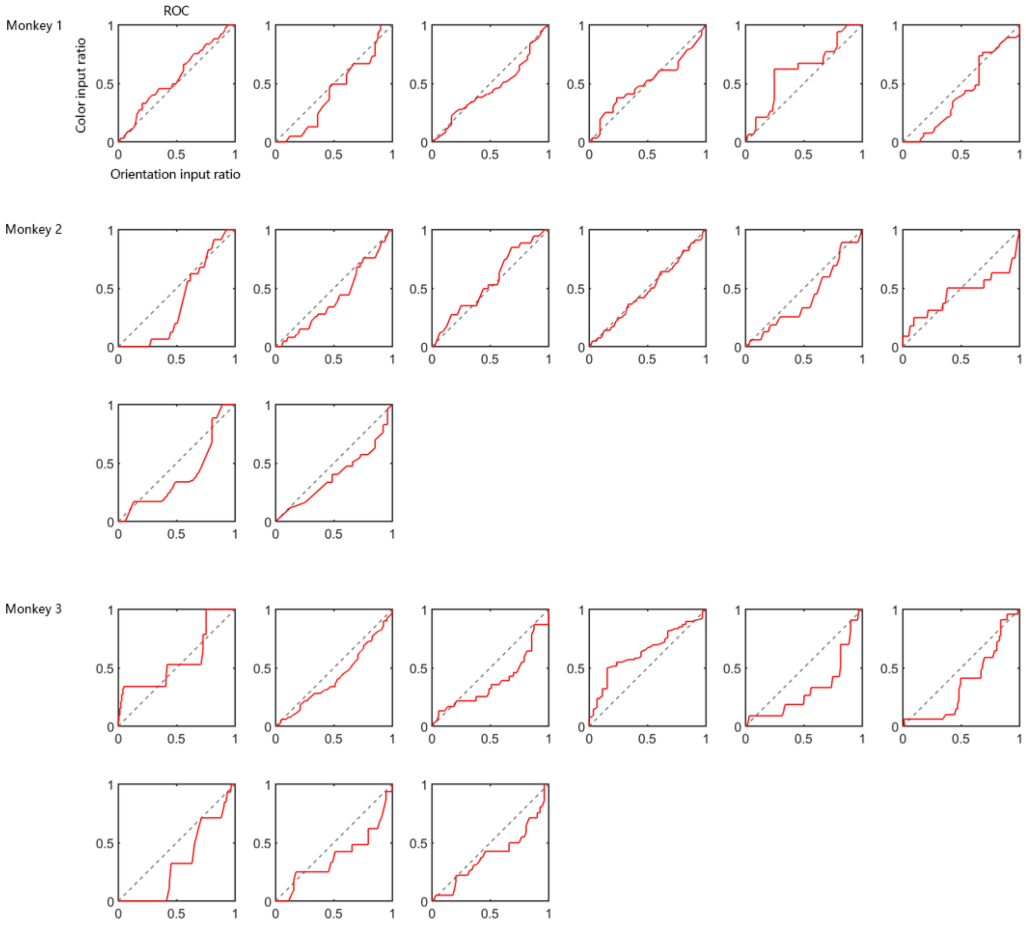

Supplementary Figure 6. Hybrid distributions of orientation-selective and color-selective inputs on neuronal dendrites. ROC analysis of synaptic input type distribution along dendrites in each neuron (AUC $= 0.45 \pm 0.09$, area under curve, mean $\pm$ STD among 23 neurons from 3 monkeys in total). See Methods for details.

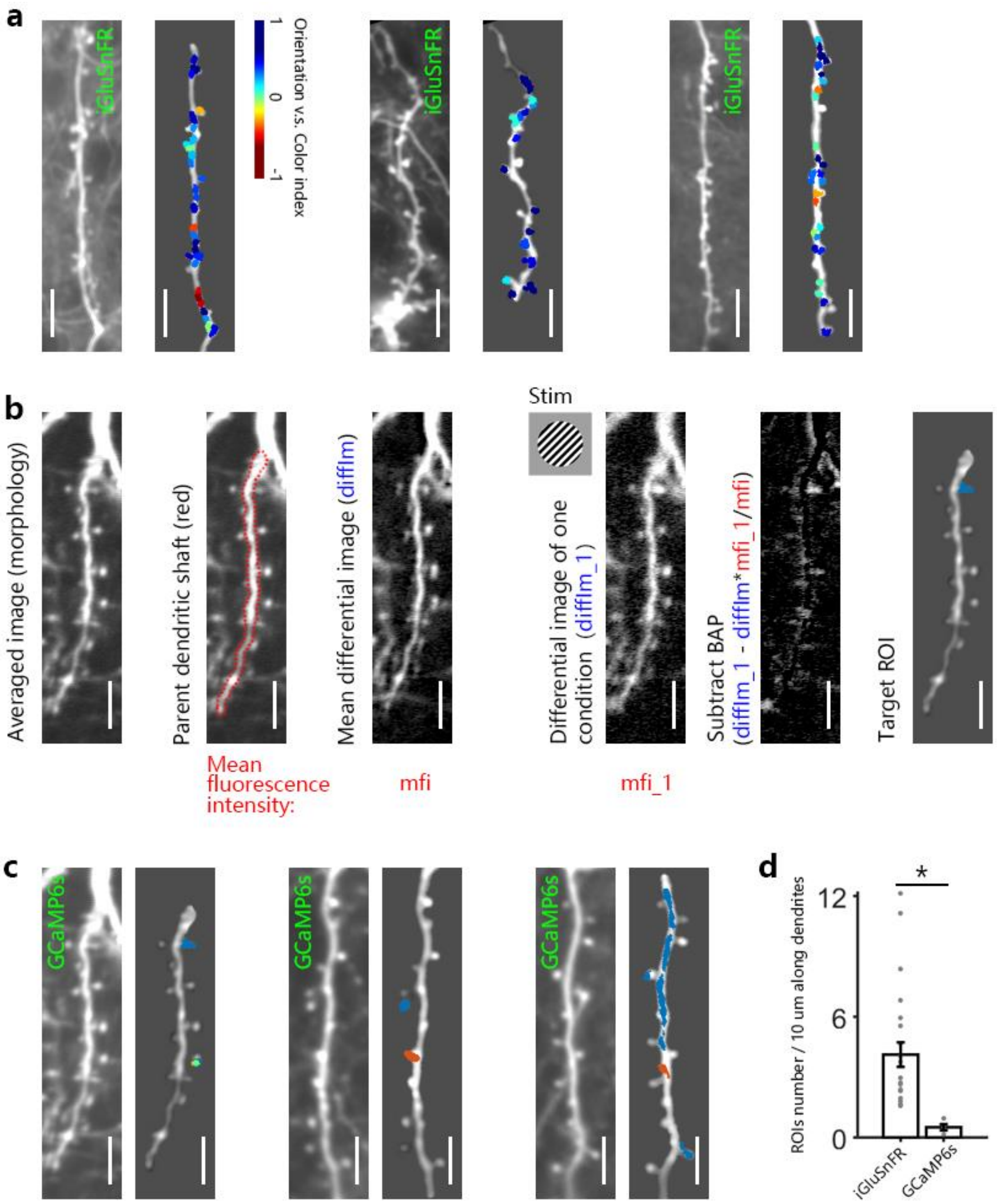

Supplementary Figure 7. Comparison between iGluSnFR and GCaMP6s recorded dendritic activities in macaques. a Dendritic shafts from neurons in superficial layer of V1 (M1N5, M2N2 and M3N5 respectively) expressing SF-iGluSnFR.A184S, with visually stimulated evoked ROIs indicated. ROIs are colored for the orientation versus color index (refer to colorbar at the upper right of the first pannel). Scale bar, 10 μm. **b** We employed a subtraction

procedure to minimize contamination from back-propagation action potentials (BAPs) during Ca-imaging of GCaMP6s responses. Leftmost, a segment of dendrite labeled with GCaMP6s, averaged from 1000 frames, recorded at a depth of 206 μm. To measure the global BAP induced dendritic signal we first outlined the parent dendritic shaft (red). Second, we obtained a mean differential image 'diffIm' across all stimulus conditions as a reference of the overall response pattern. 'mfi' represented the mean fluorescence intensity of outlined region. Third, we generated a differential image 'diffIm_1' from each specific stimulus condition, assigning the mean fluorescence intensity of dendritic region to 'mfi_1'. We then subtracted the global BAP signal and measured the fluorescence changes on the target dendrites by using the equation: diffIm_1 – diffIm*mfi_1/mfi. Rightmost, target ROI responding to this stimulus condition. **c** Three samples of dendritic shafts from neurons expressing GCaMP6s, with their corresponding evoked ROIs. **d** Density comparison of recorded ROIs on iGluSnFR and GCaMP6s expressing neural dendrites.

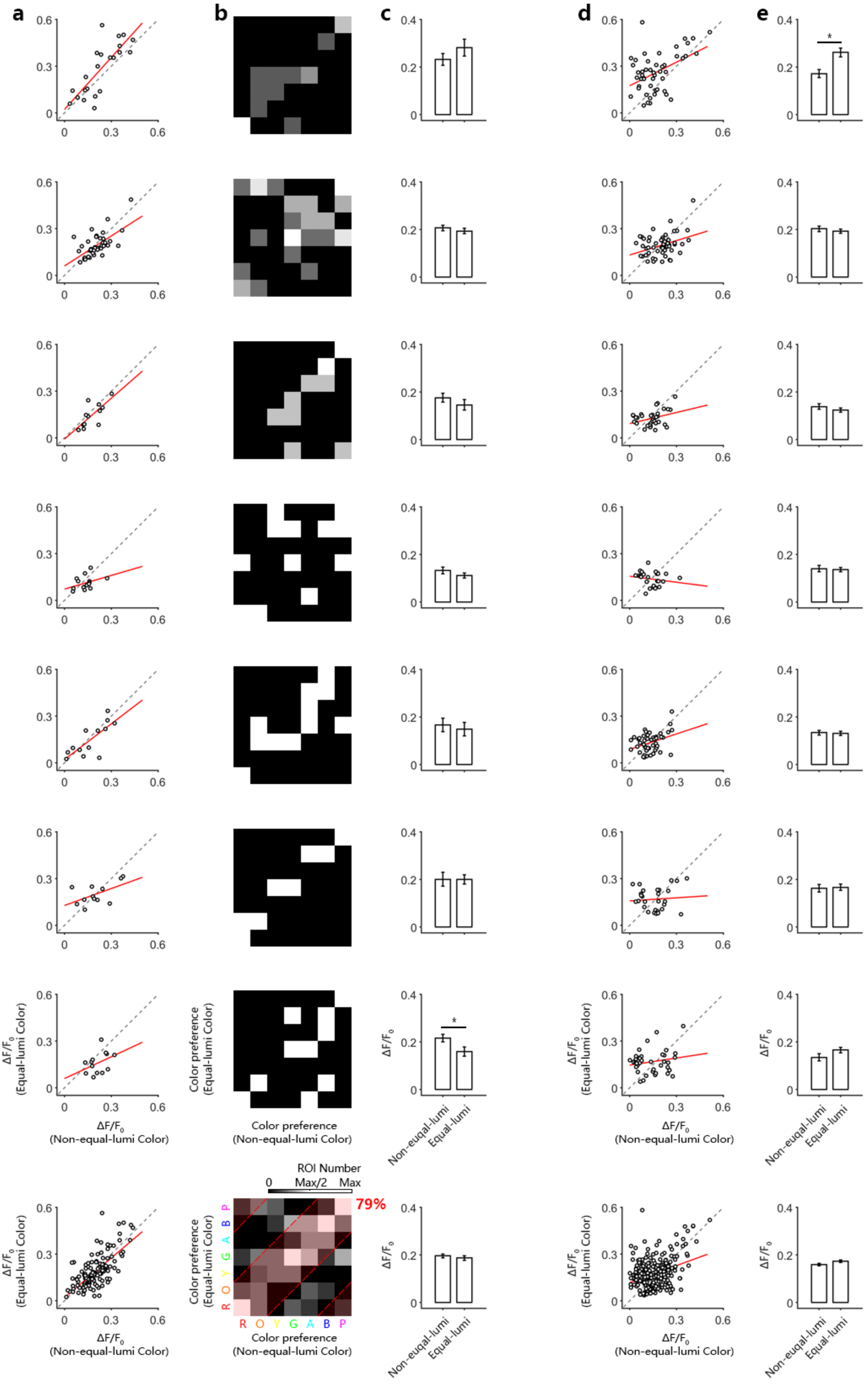
a
b
c
d
e
ΔF/F0 (Equal-lumi Color)
ΔF/F0 (Non-equal-lumi Color)
Color preference (Equal-lumi Color)
Color preference (Non-equal-lumi Color)
ROI Number
0
Max/2
Max
79%
R O Y G A B P
ΔF/F0
Non-euqal-lumi
Equal-lumi
*

Supplementary Figure 8. Comparison between non-equiluminant and equiluminant chromatic stimulus evoked responses. a Peak response intensities of ROIs to equiluminant color stimuli versus non-equiluminant (but colored) stimuli. Red line, linear regression. 7 neurons in total from Monkey 3 were recorded. Bottom, statistics for all ROIs from all 7 neurons. **b** Color preference of each ROI among equal-luminance colors versus non-equal-luminance colors. Brightness denotes ROI proportion, referring to colorbar above the bottom figure. **c** Averaged response intensity of all ROIs under non-equiluminant versus equiluminant chromatic stimuli. ROIs in **a-c** were collected only according to non-equiluminant color stimuli. Asterisk, significant difference, by Wilcoxon rank sum test; $p = 0.11$ when combining all ROIs. **d** Non-equiluminant and equiluminant chromatic stimuli response strengths within ROIs that were tested with both. **e**, Same as **c**, statistics of averaged response intensities; $p = 0.07$ when combining all ROIs.
