# Supplementary material for: Spatiotemporal functional organization of excitatory synaptic inputs onto macaque V1 neurons
Source: Nat Commun. 2020 Feb 4;11:697. doi: 10.1038/s41467-020-14501-y (PMC7000673; doi:10.1038/s41467-020-14501-y)
Supplement: Supplementary file 4 — Description of Additional Supplementary Files [file 41467_2020_14501_MOESM4_ESM.pdf]

Description of Additional Supplementary Files

File Name: Supplementary Movie 1
Description: A representative movie of the iGluSnFR recordings, recorded at a depth of 127 µm on day 71 post-infection. In this sample movie, the visual stimuli are indicated at the top-left corner. Each visual stimulus was presented for 0.5 s with a 1 s inter-stimulus interval. Frames in the movie were differential images (baseline fluorescence subtracted) averaged across 21 trials. Robust and spatially localized fluorescence increases were evoked on dendrites. Although there were some strong signals outside target neuron dendrites, such signals could be reliably distinguished as occurring on neighboring dendrites or axons.

File Name: Supplementary Movie 2
Description: Another representative movie of the iGluSnFR recordings, recorded at a depth of 105 µm on day 88 post-infection.
